# Married women’s decision to delay childbearing, and loneliness, severe psychological distress, and suicidal ideation under crisis: online survey data analysis from 2020 to 2021

**DOI:** 10.1186/s12889-023-16476-z

**Published:** 2023-08-28

**Authors:** Midori Matsushima, Hiroyuki Yamada, Naoki Kondo, Yuki Arakawa, Takahiro Tabuchi

**Affiliations:** 1 Faculty of Humanities and Social Sciences, University of Tsukuba, 1-1-1 Tennodai, Tsukuba Ibaraki, 305-8577 Japan; 2 Department of Economics, Keio University, Tokyo, Japan; 3 Department of Social Epidemiology, Graduate School of Medicine, University of Kyoto, Kyoto, Japan; 4 Departments of Health and Social Behavior, Graduate School of Medicine, University of Tokyo, Tokyo, Japan; 5 Cancer Control Center, Osaka International Cancer Institute, Osaka, Japan

**Keywords:** Loneliness, Severe psychological distress, Suicidal ideation, Pregnancy postponement, COVID-19 pandemic

## Abstract

**Background:**

The COVID-19 pandemic has affected every aspect of our lives, including the decision to become pregnant. Existing literature suggests that infertility and the decision to delay childbearing at a younger age are associated with a lower level of well-being and regrets when women start to desire a baby. Thus, the decision to delay childbearing due to the pandemic could negatively affect the well-being of women. This study focuses on how pregnancy decisions affect the well-being of women during the COVID-19 pandemic.

**Methods:**

From the Japan COVID-19 and Society Internet Survey, a nationally representative web-based survey, 768 observations of married women aged 18 to 50 years who had the intention of getting pregnant during the pre-pandemic period (conducted in 2020 and 2021) were used. Loneliness, severe psychological distress, and suicidal ideation were used as well-being indicators. For pooled data, a generalised estimated equation (GEE) model was used to estimate how pregnancy decision related to well-being indicators. For a sub-analysis, the sample was divided by the survey year and a Poisson regression model was used.

**Results:**

The GEE analysis showed an association between delaying childbearing and severe psychological distress, with the prevalence ratio (PR) being 2.06 [95% CI (1.40–3.03)]. Furthermore, loneliness and suicidal ideation that occurred after the beginning of the pandemic were significantly related to the decision to delay childbearing—1.55 [95% CI (1.03,2.34)] and 2.55 [95% CI (1.45–4.51)], respectively. Moreover, these PRs were larger for 2021 compared to 2020.

**Conclusion:**

During the COVID-19 pandemic, approximately one-fifth of married women who had childbearing intentions before the pandemic decided to postpone pregnancy. They exhibited a deteriorated mental health state. Furthermore, the negative associations were larger in 2021 compared to 2020. Loneliness has negative consequences for both mental and physical health, as well as elevated severe psychological distress and suicidal ideation among those who decided to postpone pregnancy. Therefore, the current results should not be overlooked by society.

## Background

Decisions to delay or forgo childbearing during the COVID-19 pandemic have been reported in several countries. Naito and Ogawa [1] revealed that the government’s request to the public stay home decreased the number of pregnancies by 5–8% during the pandemic in Japan. Micro-level evidence was reported in Italy, Germany, France, Spain, and the U.K. in 2021 [2] as 37.9%, 55.1%, 50.7%, 49.6%, and 57.8%, respectively, claimed to have postponed their pregnancy. Evidence was also found in China; 33.8% of couples with pregnancy intentions during the pre-pandemic period decided to cancel their pregnancy plans during the pandemic [3]. Japan is not an exception as Matsushima et al.[4] reported that approximately 20% of the married women who had pregnancy intentions during the pre-pandemic period postponed their pregnancy due to the pandemic-related factors such as a decline in income and anxiety about future household finances. Therefore, these decisions were made as a result of the COVID-19 pandemic. If these decisions have made women's health deteriorated, this needs to be addressed as a public health concern.

Most previous studies on well-being related to pregnancy focused on infertility; there is consensus that infertility lowers the level of well-being, particularly for women [5–8] and successful pregnancy leads to an improvement in well-being [9, 10]. Some studies focused on people with infertility treatment and revealed that women regret their decision to delay childbearing while they were younger and that it leads to a low level of well-being [11, 12]. Cooke et al. [11] claim that a complex interplay of factors outside of women’s control and/or conscious choice determines childbearing delay. Furthermore, according to Bunting et al. [13], a lack of fertility knowledge, including age-related infertility and risk factors of infertility, contributes to the childbearing delay decision, leading to regret at an older age, to which Japanese women are not an exception [12].

Given the above previous literature, the potential deterioration of the well-being of women who decided to postpone their pregnancy due to COVID-19 is a concern. Although previous studies explored associations between well-being and infertility and regrets over the decision to delay childbearing of women who desired a child in later life, they were conducted in fertility clinics with a small number of observations. There has been no study investigating how the decision to delay childbearing is associated with the well-being of the general population. Furthermore, the well-being indicators used in previous studies are limited to life satisfaction and regrets. Therefore, this study aims to enhance the understanding of the well-being of women by utilising a large Japanese web-based survey targeting the general population with 768 observations. In addition, examining loneliness, severe psychological distress, and suicidal ideation can provide more insights into how the decision to delay childbearing is associated with well-being.

## Methods

### Study design and participants

We used data from two rounds of a population-based online questionnaire survey, the Japan COVID-19 and Society Internet Survey (JACSIS), complying with the ethical standards of the relevant national and institutional committees on human experimentation and the 1975 Declaration of Helsinki and its 2008 revision. Ethical approval for the research protocol was obtained from the Research Ethics Committee of [The Research Ethics Committee of the Osaka International Cancer Institute] (approved 19 June 2020; approval no. 20084). The Internet survey agency adhered to the Act on the Protection of Personal Information in Japan. The participants received credit points called ‘Epoints’ that could be used for online shopping and cash conversion. These datasets were not deposited in a public repository because of confidentiality issues and the restrictions imposed by the ethical committee.

A detailed JACSIS study design has been documented by Miyawaki et al. [14]. A large number of studies have been published related to COVID-19 by using this survey including topics related to loneliness and well-being such as Taniguchi et al. [15] and Yamada et al. [16]. Among them, our study is closely related to Tachikawa et al. [17], and Matsushima et al. [4] in terms of well-being indicators, and target population with the focus on pregnancy postponement, respectively. Also, we used a methodology in common. The first survey was conducted from 25 August 2020 to 30 September 2020, with a target sample size of 28,000 individuals. A total of 224,389 panellists (men and women aged 15–79 years) were invited using random sampling stratified by sex, age, and prefecture to cover Japan based on the 2019 population distribution. The survey was closed when the target number was reached. The second-round survey was conducted from 8 to 26 February 2021. This was a follow-up to the first-round survey and was completed by 24,059 of 28,000 participants. A total of 1,941 new participants were recruited using the same sampling technique that was used in the first-round survey. In total, 26,000 samples were obtained.

Exclusion criteria were established to maintain the quality of the data. First, responses with discrepancies and/or unusable answers were excluded, leaving 25,482 (2020 data) and 23,142 (2021 data) samples. The exclusion criteria were: (1) an invalid response to ‘Please choose the second alternative from the bottom’ (i.e. the panellists who failed to select the second last alternative from the five options available; this question was included to identify systematic respondent inattention); (2) positive responses to all questions related to drug use (e.g. marijuana, cocaine, or heroin); and (3) positive answers to all questions regarding 16 underlying chronic diseases. We further excluded respondents who were male (*n* = 12,673 [2020], *n* = 11,766 [2021]), aged < 18 or > 50 years (*n* = 6,134 [2020], 5,736 [2021]), unmarried or widowed (*n* = 3,323 [2020], *n* = 2,828 [2021]), with no plan of pregnancy (*n* = 2,851 [2020] and 2,391 [2021 data]), and with incomplete responses to other variables (*n* = 82 [2020], *n* = 73 [2021]). Finally, we employed 768 observations, with 420 and 348 observations in 2020 and 2021, respectively. Thus, these two rounds of the survey enabled us to create partial panel data set including 202 of 348 observations in 2021 which are the follow-up ones of the first-round survey.

### Measures

#### Loneliness

Two indicators of loneliness were used: The first indicator was the University of California, Los Angeles (UCLA) Loneliness Scale Version 3, Short Form 3-item (UCLA-LS3-SF-3), the latest version of the three-item short form (5-point Likert scale ranging from 1 [*never*] to 5 [*always*]), which was validated by Arimoto and Tadaka [18] in Japan. The questions were (in the past 30 days): (1) How often do you feel that you lack companionship? (2) How frequently do you feel left out? (3) How often do you feel isolated from others? The total score ranged from 3–15. This study followed Yamada et al.’s [16] strategy, also adopted by Tachikawa et al. [17] to define ‘moderate-to-severe loneliness’ as a total score of 6–15. A dummy variable was set as 1 if the results indicated moderate-to-severe loneliness and 0 otherwise.

The second indicator, measured on a 5-point scale ranging from 1 (*never*) to 5 (*always*), answered the question: ‘Do you think you have experienced loneliness more frequently recently than before the COVID-19 pandemic?’ A dummy variable was set where 1 = feeling lonely more often due to COVID-19 (for scores of 4 or 5) and 0 = not feeling lonely (for scores of 1, 2, or 3).

### Severe psychological distress

The Kessler Psychological Distress Scale (K6), a 6-item questionnaire developed for screening mood and anxiety disorders by asking about the experience of the past 30 days [19], was used to measure depression. Total K6 scores ranged from 0 to 24, with higher scores indicating more severe distress. The Japanese version was validated by Furukawa et al. [20], and K6 ≥ 13 was adopted as the cut-off value, indicating a severe psychological distress.

### Suicidal ideation

Suicidal ideation was measured by the question, ‘Have you ever felt that you wanted to die since April 2020?’ The responses were chosen from: ‘1. Yes, I felt it for the first time’ 2. ‘Yes, I felt it before April 2020’ and ‘3. No, I did not have it’. Using this, the following two variables were created: The first was ‘Presence of suicidal ideation’ (1 = If the respondent chose either 1 or 2 from the above choices and 0 = otherwise; i.e. having suicidal ideation since the COVID-19 pandemic). The second variable attempted to capture the onset of suicidal ideation during the COVID-19 pandemic and was constructed as 1 = if the respondent chose 1 from the above choices and 0 = if the respondent chose 3. The same strategy as Tachikawa et al. [17] was used for this variable construction.

### Exposures

#### Decision to delay childbearing

The participants were asked, ‘In the past two months, have you avoided becoming pregnant, despite your plan to be pregnant, due to COVID-19?’ Answers comprised the following three options: 1 (*yes*), 2 (*no*), and 3 (*not applicable; no plans for pregnancy*). Data from respondents who answered either 1 (*yes*) or 2 (*no*) were used. Although 3 (*not applicable*) contained important information, such as ‘not wanting a child anymore’ or ‘cannot have a child due to fertility issues’, whether the respondents happily prevented their childbearing or disappointedly gave it up could not be distinguished. Therefore, this research excluded the respondents who chose ‘3’. This variable is created based on Matsushima et al. [4].

### Covariates

This study included social isolation as it has been used as a variable to predict deterioration of well-being, COVID-19-related indicators, and socio-demographic characteristics. First, we included social isolation indicators, defining social isolation as ‘less than once per two weeks of social contact’, and having a child or not. For COVID-19-related indicators, income decline, anxiety regarding household financial outlook, fear of COVID-19, and the number of COVID-19 positive cases in the residential province were used. Income, home ownership, employment status, age, and educational attainment were included to control for demographic characteristics. Moreover, whether the patient was undergoing fertility treatment was also included. These covariates were commonly used in Matsushima et al. [4].

### Analytical model

First, we used a generalised estimated equation (GEE) model with the 2020 and 2021 data, assuming a Poisson distribution, and estimated prevalence ratios (PRs) with robust standard errors. This model, a quasi-likelihood method based on generalised linear models, was employed because the dataset is partial panel data and the outcomes are binary variables [21, 22]. Goodness-of-fit chi-square tests were conducted for each analysis, and the results were not statistically significant. Subsequently, the 2020 and 2021 data were analysed separately using the Poisson regression model with robust standard errors to observe any changes in the associated factors. Sampling weights were applied to each of the datasets.

## Results

First, the results of the chi-square test and t-test are also shown for categorical and continuous variables, respectively, to observe differences in characteristics between women with and without the intention to postpone their pregnancy with approximately 20% of married women postponing pregnancy (Table 1). A lower level of well-being was indicated for those who postponed their pregnancy. Among those who postponed their pregnancy during the pandemic, over 50% felt moderate-to-severe loneliness, about 32% were having severe psychological distress, and about 29% had suicidal ideation. Moreover, about 28% and 20% were feeling more lonely and experiencing to have suicidal ideation for the first time after the pandemic. Among respondents who did not postpone their pregnancy, about 33% felt moderate-to-severe loneliness, about 12% were having severe psychological distress, and about 17% had suicidal ideation. Also, the percentage of people who were feeling more lonely and experiencing to have suicidal ideation for the first time after the pandemic were less than half and about one-fifth of those who postponed pregnancy (Table 1, Column 1). The gap between those two groups increased in 2021 except with regard to suicidal ideation (Table 1, Columns 2 and 3). Demographic characteristics of respondents between the ones postponed pregnancy and ones who did not were indifferent except for age, fertility treatment, and being a property owner.

**Table 1:** Characteristics of participants

	(1) Total (*n* = 768)							(2) 2020 (*n *= 420)							(3) 2021 (n = 348)						
	*Pregnancy postpone*							*Pregnancy postpone*							*Pregnancy postpone*						
	Yes (*n* = 153)			No (*n *= 615)				Yes (*n* = 88)			No (*n* = 332)				Yes (*n *= 65)			No (*n *= 283)			
	*n*	%	Mean (SD)	*n*	%	Mean (SD)	*P*- value	n	%	Mean (SD)	n	%	Mean (SD)	*P*- value	n	%	Mean (SD)	n	%	Mean (SD)	*P*- value
***Well-being indicators***																					
***UCLA Loneliness Scale***							< 0.001							0.002							0.009
***Moderate-to-severe loneliness***	77	50.33		204	33.17			41	46.59		97	29.22			36	55.38		107	37.81		
***None or mild loneliness***	76	49.67		411	66.83			47	53.41		235	70.78			29	44.62		176	62.19		
***Psychological distress***							< 0.001							< 0.001							< 0.001
***K6* > = *13***	49	32.03		73	11.87			27	30.68		35	10.54			22	33.85		38	13.43		
***K6* < *13***	104	67.97		542	88.13			61	69.32		297	89.46			43	66.15		245	86.57		
***Suicidal ideation***							0.001							< 0.001							0.348
***Yes***	44	28.76		103	16.75			29	32.95		52	15.66			15	23.08		51	18.02		
***No***	109	71.24		512	83.25			59	67.05		280	84.34			50	76.92		232	81.98		
***Well-being indicators (deterioration after the onset of the pandemic)***																					
***Loneliness after COVID-19***							< 0.001							< 0.001							0.001
***Yes***	43	28.10		69	11.22			25	28.41		37	11.14			18	27.69		32	11.31		
***No***	110	71.90		546	88.78			63	71.59		295	88.86			47	72.31		251	88.69		
***Suicidal ideation after COVID-19***							< 0.001							< 0.001							< 0.001
***Yes***	30	19.61		27	4.39			19	21.59		16	4.82			11	16.92		11	3.89		
***No***	123	80.39		588	95.61			69	78.41		316	95.18			54	83.08		272	96.11		
*Isolation*							0.109							0.198							0.267
*Yes*	9	5.88		62	10.08			7	7.95		43	12.95			2	3.08		19	6.71		
*No*	144	94.12		553	89.92			81	92.05		289	87.05			63	96.92		264	93.29		
*Currently having no child*							< 0.001							< 0.001							0.144
*Yes*	61	52.94		224	36.42			51	57.95		121	36.45			30	46.15		103	36.40		
*No*	92	47.06		391	63.58			37	42.05		211	63.55			35	53.85		180	63.60		
*Income decline*							< 0.001							0.001							0.027
*Yes*	76	49.67		199	32.36			49	55.68		121	36.45			27	41.54		78	27.56		
*No*	77	50.33		416	67.64			39	44.32		211	63.55			38	58.46		205	72.44		
*Feeling anxious about household financial outlook*							< 0.001							< 0.001							0.001
*Yes*	81	52.94		197	32.03			48	54.55		113	34.04			33	50.77		84	29.68		
*No*	72	47.06		418	67.97			40	45.45		219	65.96			32	49.23		199	70.32		
*Fear of COVID-19 (score 7–35)*			19.50 (5.91)			18.96 (5.81)	0.306			19.61 (5.84)			18.79 (5.90)	0.242			19.35 (6.05)			19.17 (5.70)	0.820
*Log of number of COVID-19 positive cases in the residential province*			7.69 (1.92)			7.75 (1.94)	0.354			6.84 (1.70)			6.93 (1.86)	0.706			8.83 (1.60)			8.72 (1.53)	0.614
*Income:*																					
*The poorest quartile (Reference)*	39	25.49		140	22.76		0.475	20	22.73		75	22.59		0.979	19	29.23		65	22.97		0.287
*The 2nd poorest*	38	24.84		161	26.18		0.735	24	27.27		105	31.63		0.431	14	21.54		56	19.79		0.751
*The 2nd richest*	40	26.14		155	25.20		0.811	24	27.27		73	21.99		0.296	16	24.62		82	28.98		0.481
*The richest*	36	23.53		159	25.85		0.554	20	22.73		79	23.80		0.834	16	24.62		80	28.27		0.552
*Property owner*							< 0.001														
*Yes*	49	32.03		316	51.38			24	27.27		177	53.31		< 0.001	25	38.46		139	49.12		0.121
*No*	104	67.97		299	48.62			64	72.73		155	46.69			40	61.54		144	50.88		
*Employment status:*																					
*Permanent full-time worker (Reference)*	63	41.18		220	35.77		0.215	37	42.05		112	33.73		0.147	26	40.00		108	38.16		0.784
*Contract full-time worker*	10	6.54		28	4.55		0.311	4	4.55		17	5.12		0.826	6	9.23		11	3.89		0.071
*Part-time worker*	26	16.99		153	24.88		0.039	16	18.18		83	25.00		0.18	10	15.38		70	24.73		0.106
*Not working*	54	35.29		214	34.80		0.908	31	35.23		120	36.14		0.873	23	35.38		94	33.22		0.739
*Age*			32.73 (5.76)			36.50 (7.40)	< 0.001			32.38 (6.10)			36.32 (7.30)	< 0.001			33.22 (5.28)			36.70 (7.52)	0.001
*Education:*																					
*High school or lower (Reference)*	19	12.42		130	21.14		0.015	14	15.91		72	21.69		0.232	5	7.69		58	20.49		0.016
*Vocational training school/2-years college*	47	30.72		201	32.68		0.642	25	28.41		108	32.53		0.46	22	33.85		93	32.86		0.879
*University or higher*	87	56.86		284	46.18		0.018	49	55.68		162	45.78		0.098	38	58.46		132	46.64		0.086
*Fertility treatment*							< 0.001							< 0.001							< 0.001
*Yes*	49	32.03		448	72.85			28	31.82		241	72.59			21	32.31		207	73.14		
*No*	104	67.97		167	27.15			60	68.18		91	27.41			44	67.69		76	26.86		

Notes for Table 1

1) Because JACSIS 2020 and 2021 did not have the same social isolation indicators, the social isolation variables constructed for this study were as follows: For 2020, the overall frequency of social contact in the survey month was calculated based on the methodology used in a previous study [20, 21]. Social contact was identified by the following eight questions: (1) ‘How often did you meet your family members or relatives who were not living with you?’ (2) ‘How often did you meet your friends?’ (3) ‘How often did you make contact with your family members or relatives who were not living with you through text messages?’ (4) ‘How often did you make contact with your friends who were not living with you through text messages?’ (5) ‘How often did you connect with your family members or relatives who were not living with you through voice calls?’ (6) ‘How often did you make contact with your friends who were not living with you through voice calls?’ (7) ‘How often did you communicate with your family members or relatives who were not living with you through video calls?’ and (8) ‘How often did you make contact with your friends who were not living with you through video calls?’ These questions could be responded using the following alternatives: almost daily (six to seven times weekly), four to five times weekly, two to three times weekly, once weekly, two to three times monthly, monthly and rarely. Because the average number of weeks in a month is 4.35 (= 365 days in a year/12 months in a year/7 days in a week), the frequency was calculated as 28.28 days per month if the respondent answered ‘almost every day (six to seven times weekly)’. Using the same method, the answers were converted into 19.58, 10.88, 4.35, 2.5, 1 and 0 days for the answer of ‘4–5 times weekly’, ‘2–3 times weekly’, ‘weekly’, ‘2–3 times monthly’, ‘monthly’ and ‘rarely’, respectively. After calculating the frequency for each question, all the questions were summed and socially isolated people were identified as those who had less than once per two weeks of social contact. For the 2021 data, the answer to the following questions were employed: ‘Who have you met or talked to more than once in the past two weeks?’ and ‘Who have you communicated through online tools more than once in the past two weeks?’ An individual was identified as socially isolated when he/she responded ‘none’ to both of these questions, excluding partners, child(ren) and colleagues. By doing this, data for 2020 and 2021 were made compatible. Murayama et al. [23] identified a socially isolated person as one who made contact less than once every week based on the evidence provided by Saito et al. [24] that having social contact less than once a week is associated with greater risks of all-cause mortality, dementia and disability. This was modified to ‘less than once per two weeks of social contacts’ in order to make it compatible with the JACSIS 2021 data. Therefore, the social isolation indicators captured even more severe cases of isolation

2) Fear of COVID-19 was measured using the Fear of Coronavirus-19 Scale (FCV-19S), developed by Ahorsu et al. [25] and validated in Japan [26]. Total scores range from 7 to 35, with higher scores indicating stronger anxiety and fear (cut-off values have not been determined thus far)

3) The number of positive COVID-19 cases in the residential province was the accumulated number of new cases for two months, including the survey months, obtained from the Kyushu Economic Research Centre [27]. The number of cases was limited to those reported during the two months around each survey period to estimate the period that we specified in our question on childbearing delay

4) The income variable was created by using the whole sample of JACSIS data for each year, excluding the ones with discrepancies and/or artificial/unnatural responses. We first calculated equivalent income, then divided it into quartiles

5) Employment status was categorised as: 1: permanent full-time worker, 2: contract/temporary full-time worker, 3: part-time worker and 4: not in labour force. Although unemployment is an important indicator, its rate in Japan is low; the current data did not contain a sufficient number to be examined. Moreover, during the COVID-19 pandemic, people may not search for jobs. Thus, the unemployed people, homemakers, and students were combined into the category of ‘4’

6) Regional dummies were included in the analysis but are not shown here for brevity. The regions were divided into eight regions; Hokkaido, Tohoku, Kanto, Chubu, Kinki, Chugoku, Shikoku, and Kyushu/Okinawa

In the results of the regression analyses, all the COVID-19 related variables and demographic characteristics were adjusted. The GEE regression results suggest that the PR of moderate-to-severe loneliness was 1.1 [95% CI (0.93–1.30)], and suicidal ideation was 1.039 [ 95% CI (0.76–1.42)]. The PR of being depressed was highest at 2.06 [95% CI (1.40–3.03)]. The Poisson regressions for each year indicated a stronger association in 2021 compared to 2020 (Tables 2 and 3).

**Table 2:** Pregnancy postponement and well-being (loneliness, severe psychological distress and suicidal ideation)

	*UCLA Loneliness scale*			*Severe psychological distress*			*Suicidal ideation*		
	*(1: Moderate-to-severe loneliness,* *0: No loneliness)*			*(1: K6* > = *13 0: K6* < *13)*			*(1: Presence of suicidal ideation,* *0: No presence of suicidal ideation)*		
	*GEE*	2020	2021	*GEE*	2020	2021	*GEE*	2020	2021
*Pregnancy postpone*	1.1	0.91	1.21	2.06^***^	1.68	2.69^**^	1.04	0.79	1.55
	[0.93,1.30]	[0.68,1.22]	[0.95,1.54]	[1.40,3.03]	[0.88,3.21]	[1.49,4.85]	[0.76,1.42]	[0.45,1.38]	[0.82,2.94]

NOTE:

1. Robust standard errors were used for all analyses. 95% confidence intervals (95% CI) are reported in square brackets

2. Prevalence ratios (PRs) are reported, and * *p* < 0.05, ** *p *< 0.01 and *** *p* < 0.001

3. Covariates listed in Table 1, and regions and survey year (for GEE analysis) were adjusted. Not reported for brevity

**Table 3:** Pregnancy postponement and deterioration of well-being during the pandemic

	*Loneliness after COVID-19*			*Suicidal ideation after COVID-19*		
	*(1: Feeling lonely more often due to COVID-19,* *0: Not feeling lonely)*			*(1: Onset of suicidal ideation after COVID-19,* *0: No onset of suicidal ideation)*		
	GEE	2020	2021	GEE	2020	2021
*Pregnancy postpone*	1.55^*^	1.55	2.37^**^	2.55^***^	2.31^*^	4.14^*^
	[1.03,2.34]	[0.73,3.26]	[1.38,4.05]	[1.45,4.51]	[1.152,4.629]	[1.4,12.29]

NOTE:

1. Robust standard errors were used for all analyses. 95% confidence intervals (95% CI) are reported in square brackets

2. Prevalence ratios (PRs) are reported, and * p < 0.05, ** p < 0.01 and *** p < 0.001

3. Covariates listed in Table 1, and regions and survey year (for GEE analysis) were adjusted. Not reported for brevity

Moreover, the decision to delay childbearing increased the PRs of loneliness and suicidal ideation after the onset of the pandemic, with a PR of 1.55 [95% CI (1.03–2.34)] and 2.55 [1.45–4.51], respectively. Sub-analysis shows that, for loneliness, the effects were larger in 2021 (PR 2.37; 95% CI [1.38–4.05]) compared to 2020 (PR 1.55; 95% CI [0.73–3.26]). The same trend was observed for the onset of suicidal ideation, with a larger PR observed in 2021 (PR 4.14; 95% CI [1.4–12.29]) compared to 2020 (PR 2.31; 95% CI [1.15–4.63]) in 2020.

## Discussion

The main finding of this study is that those who decided to postpone pregnancy were found to have a higher PR of having severe distress, more frequent loneliness after the pandemic, and the onset of suicidal ideation for the first time during the pandemic. It was also found that COVID-19 related factors, such as income decline, anxiety towards household financial outlook, and fear of COVID-19, are associated with women’s well-being. Besides, when these negative events were held constant, a significant adverse association between pregnancy delay and well-being was still observed.

This study used K6 to capture the respondents’ distress and adopted the cut-off point of 13 to identify people with the state of severe psychological distress [19, 23]. The results suggested that PR was more than two-folds among the women with pregnancy delays, and the effect was stronger in 2021. For loneliness, the decision to delay childbearing increased the PR of the more frequent incidence of feeling lonely after the onset of the pandemic, and the effects were larger in 2021 compared to 2020. Loneliness occurs when one experiences a gap between actual and desired levels of social engagement [28]. If a woman desires to become pregnant but decides not to do so due to the pandemic, she may be overwhelmed by the discrepancies between her expectations and desires for having a child and her current decision to postpone childbearing. Another finding that should be noted is the increase in suicidal ideation among those who postponed their pregnancy both in 2020 and 2021. In particular, in 2021, more than four times the normal PR of suicidal ideation was indicated. Possible reasons for the stronger effects of pregnancy postpone on the more frequent incidence of loneliness and suicidal ideation after the onset of the pandemic are the prolonged pandemic that caused the loss of hope to be pregnant in future or escalating anxiety not knowing how long this situation last. According to Branley-Bell et al. [29], depression severity, feelings of defeat and entrapment, and/or loneliness have been found to lead to suicidal thoughts and attempts.

Taking the results of other well-being indicators into consideration, this elevation in suicidal ideation may lead to an increment in the number of suicide deaths. There have been continuous reports of excess suicide deaths among women in Japan since the pandemic, although it is not the case for men. Nomura et al. [30] found excess suicide deaths among women in July, August, and September 2020, followed by Yoshioka et al. [31] for the period of April 2020 to December 2021, and Batista et al. [32] for the period of March 2020 to April 2022. According to Koda et al. [33], women were more likely to be influenced by relationships with family members, including marital discord and infidelity. Pregnancy postponement could be a cause and consequence of marital discord and it could indirectly affect women’s well-being through marital discord. Another possible path could be that the pandemic has led to marital discord and pregnancy postponement, which deteriorated women’s well-being. In either case, the respondents reported that their decisions to delay childbearing were due to the COVID-19 pandemic, implying that this pandemic has induced some behavioural changes and is associated with a higher prevalence of depression, loneliness, and suicidal ideation. The current results are consistent with previous studies conducted in fertility clinics [11, 12] and also prove that they are applicable to the general public.

This study has some limitations. First, the study sample was obtained through an online survey. Although the sampling methods strived to ensure representativeness by employing random sampling techniques stratified by sex, age, and prefecture to cover Japan based on the 2019 population distribution, the target population comprised those who were planning to become pregnant up until the pandemic. Thus, the representativeness of the survey does not necessarily ensure the representativeness of this particular population. Moreover, the possible bias due to the nature of the online survey has not been eliminated. In addition, due to the long length of the survey, women with severe mental health problems might not be able to complete the survey, leading to an underestimation of the current results. Second, this study cannot determine causality. Furthermore, it could not be specified exactly when they started to feel lonely and/or depressed and have suicidal ideation. It could be the case that they have been in the same state of well-being since the pre-pandemic period and might have therefore chosen to postpone pregnancy. Although the regression results using the original indicators of worsened loneliness and onset of suicidal ideation after the pandemic showed that childbearing delay and deterioration of mental health occurred during the pandemic, it cannot identify causality because the worsened loneliness and the onset of suicidal ideation after the pandemic may have influenced the decision to postpone childbearing.

## Conclusions

During the COVID-19 pandemic, around one-fifth of married women who had childbearing intentions before the pandemic decided to postpone their pregnancy. This study showed that those who were delaying their pregnancy had a deteriorated state of well-being. Furthermore, the negative association was larger in 2021 compared to 2020. It is a well-investigated fact that loneliness has negative consequences for both mental and physical health, and there already was elevated psychological distress and suicidal ideation among those who decided to postpone pregnancy, which should not be overlooked in society. Besides, lessons learnt from the experiences during the COVID-19 pandemic have implications for the future as we may face another crisis exogenously leading to childbearing delay. The current findings recommend making preparations to promptly provide mental care in maternal health services to prevent an escalation in loneliness, severe psychological distress, and suicidal ideation during a crisis.

## Data Availability

These datasets are not deposited in a public repository because of the confidentiality and restrictions imposed by the data of the ethical committee. Dr. Takahiro Tabuchi was responsible for data management. Data inquiries should be addressed by e-mail (tabuchitak@gmail.com). Questionnaire is provided in "https://jacsis-study.jp/howtouse/".

## Abbreviations

COVID-19: The coronavirus disease 2019
JACSIS: The Japan COVID-19 and Society Internet Survey
PR: Prevalence Ratio
CI: Confidence interval
UCLA-LS3-SF-3: California, Los Angeles (UCLA) Loneliness Scale Version 3, Short Form 3-item
K6: The Kessler Psychological Distress Scale
SD: Standard deviation
FCV-19S: The Fear of Coronavirus-19 Scale
